# Chicago Hospital

**Published:** 1847-04

**Authors:** 


### PART V.—EDITORIAL.

### ARTICLE I.

### CHICAGO HOSPITAL.

We have the pleasure of announcing to our readers that the public authorities have determined to establish a hospital at Chicago, and have already taken such steps as to put it in immediate operation. This measure imperiously demanded by the public interest, on the score of economy, and that of the indigent on the ground of humanity, is no less so by the interests of medical science as a means of improvement. A moment's consideration will show us the advantages it will afford in this respect. A large proportion of all the inhabitants of the western states have, during the past summer, suffered under periodical fevers. Their effects, in form of dropsical effusions, enlarged spleen, chronic phlegmasia, and debility remain. The disease itself often occurring, even during the winter, and baffling any attempt to arrest it permanently and remove its sequelæ.

The universal decision of the public and of the profession is, that all known means of treating such affections are ineffectual, and that new researches on the subject are required. These can only be made advantageously in public institutions.

During the past winter researches made in the dispensary of this city, have shown that the decoction of the bark of wahoo possesses advantages over all other substances in the treatment of dropsical effusions following intermittent and remittent fevers. More recent investigations, pursued in the same institution, have shown also, that there are articles in the materia medica which possess a far more prompt and permanent influence on those agues of long standing than quinine, arsenical preparations, iron, or bitter tonics. These, when completed, will be made public. It was necessary that opportunity should be afforded for their continuance.

In the new hospital every care will be taken to observe and record the cases, and direct the treatment so as to reap the utmost advantages which they are capable of affording.

The arrangements of the medical department of the hospital will be of the most liberal kind. It will be entrusted entirely at present to the care of Dr. Brainard, who will spare no pains to make it useful as a means of teaching as well as a benefit to the sick. The dispensary will be continued in connection with the hospital, and out doors patients will be visited at their dwellings.

Added to the means already afforded at Chicago, those we have described for clinical instruction place this city with the first in the Union in respect to the advantages afforded to medical students. Already have the anatomical students found facilities for making preparations, dissecting, preparing models of plaister, &c., not offered elsewhere.

But no system of medical education can be considered sufficient which does not embrace clinical instruction. It is for want of this that so many young men fail of gaining the confidence of the public, of treating disease successfully, of sustaining the character of scientific physicians. It is this lamentable ignorance which discredits the profession in the public mind. Thus we see so many M. D.s, and even professors, in practice, thrown into the shade by uriscopians, Thompsonians and others whom they affect to despise. It is owing to this want of practical knowledge that medical men become routinists, and administer calomel, ant. tart., quinine, and other powerful drugs indiscriminately, so that in every respect, excepting in name, they might be ranked with the quacks themselves. As anatomy and physiology form the basis, so does clinical medicine, with the accessory knowledge it requires or affords, constitute the superstructure of medical science. This may, indeed, be gained in private practice, but it can only be done by much trouble with a teacher and much danger to the public after the physician has commeuced practice on his own responsibility. But a well instructed physician will be ready to meet disease successfully as soon as he enters upon the duties of his profession.

The establishment of a hospital at Chicago is particularly gratifying to us, and will be, we trust, to our subscribers, from the additional interest it will enable us to give to the pages of this Journal. Reports of interesting cases, or the results of treatment in classes of disease will be furnished to our pages from time to time.

*Chicago, March 27th* 1847.

